# Correction to “Severe Thiopurine‐Induced Myelosuppression in a Pediatric Acute Lymphoblastic Leukemia Patient ith the NUDT15 *1/*6 Genotype: A Brief Report”

**DOI:** 10.1111/cts.70652

**Published:** 2026-06-23

J. Fry , E. C. Boone , W. Y. Wang , et al., “Severe Thiopurine‐Induced Myelosuppression in a Pediatric Acute Lymphoblastic Leukemia Patient With the NUDT15 *1/*6 Genotype: A Brief Report,” Clinical and Translational Science 19, no. 6 (2026): e70639, 10.1111/cts.70639.42286409 PMC13263158

In the article cited above, the data points in Figure 2 are not visible beneath the green and red bars.

We apologize for this error.

**Figure:**
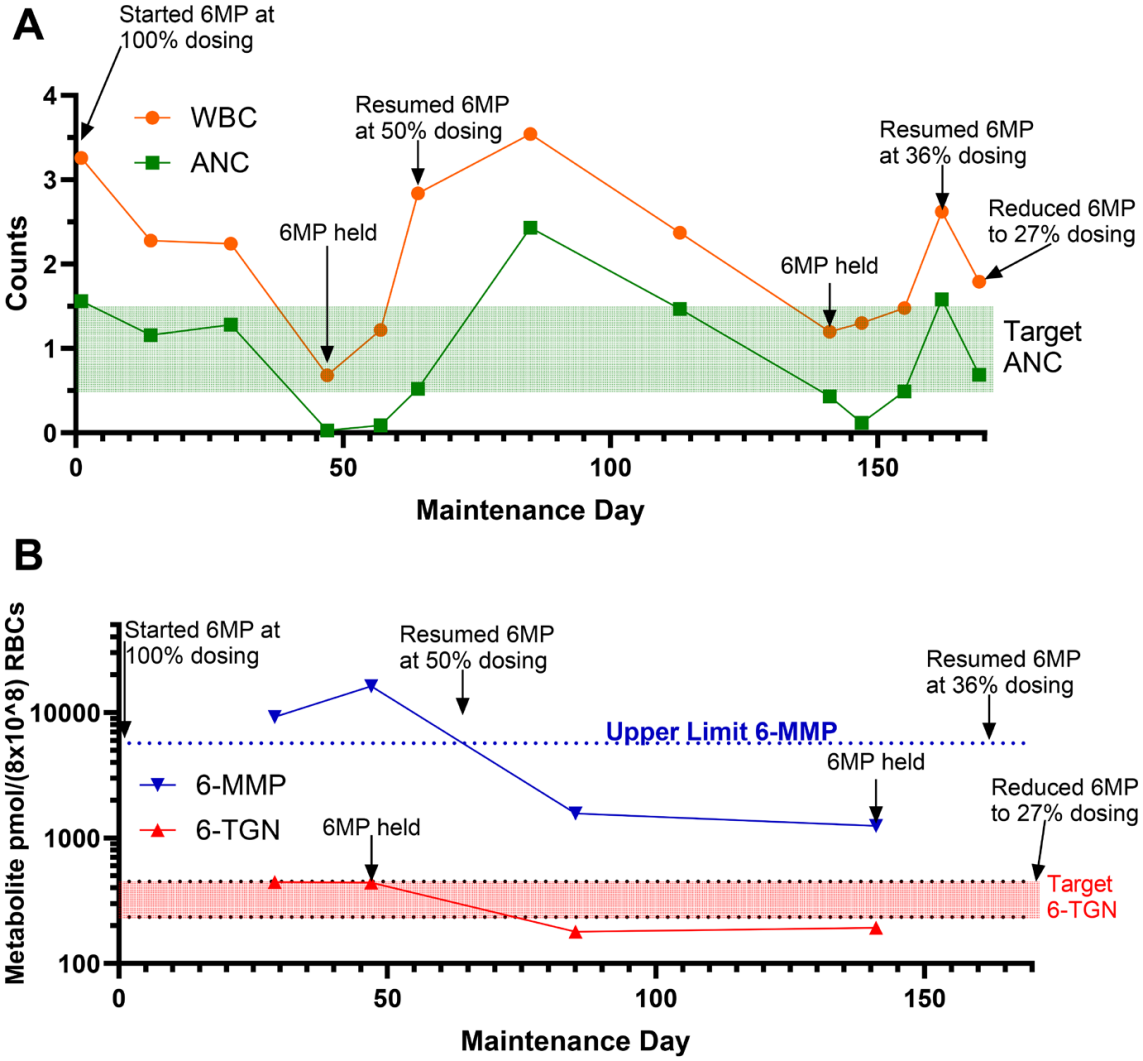
**FIGURE 2**White blood cell (WBC) count and absolute neutrophil count (ANC) in the Maintenance phase of chemotherapy with time points marked where mercaptopurine (6MP) was held, resumed, or reduced in dose (A). 6‐TGN and 6‐MMP metabolites in the Maintenance phase of chemotherapy with time points marked where mercaptopurine (6MP) was held, resumed, or reduced in dose (B). During Maintenance, 6MP dosing was adjusted based on ANC and platelet counts, per protocol.

